# Metabolomic and transcriptomic analyses reveal the effects of self- and hetero-grafting on anthocyanin biosynthesis in grapevine

**DOI:** 10.1093/hr/uhac103

**Published:** 2022-05-17

**Authors:** Haixia Zhong, Zhongjie Liu, Fuchun Zhang, Xiaoming Zhou, Xiaoxia Sun, Yongyao Li, Wenwen Liu, Hua Xiao, Nan Wang, Hong Lu, Mingqi Pan, Xinyu Wu, Yongfeng Zhou

**Affiliations:** 1 Institute of Horticulture Crops, Xinjiang Academy of Agricultural Sciences (Key Laboratory of Genome Research and Genetic Improvement of Xinjiang Characteristic Fruits and Vegetables), Urumqi, China; 2 Shenzhen Branch, Guangdong Laboratory of Lingnan Modern Agriculture, Genome Analysis Laboratory of the Ministry of Agriculture and Rural Affairs, Agricultural Genomics Institute at Shenzhen, Chinese Academy of Agricultural Sciences, Shenzhen, China

## Abstract

Grafting, which joins a scion from a cultivar with the stem of a rootstock from a grapevine wild relative, is commonly used in viticulture. Grafting has crucial effects on various phenotypes of the cultivar, including its phenology, biotic and abiotic resistance, berry metabolome, and coloration, but the underlying genetics and regulatory mechanisms are largely unexplored. In this study, we investigated the phenotypic, metabolomic, and transcriptomic profiles at three developmental stages (45, 75, and 105 days after flowering) of the Crimson Seedless cultivar (*Vitis vinifera*) grafted onto four rootstocks (three heterografts, CS/101-14, CS/SO4, and CS/110R and one self-graft, CS/CS) with own-rooted graft-free Crimson Seedless (CS) as the control. All the heterografts had a significant effect on berry reddening as early as ~45 days after flowering. The grafting of rootstocks promoted anthocyanin biosynthesis and accumulation in grape berries. The metabolomic features showed that cyanidin 3-O-glucoside, delphinidin 3-O-glucoside, malvidin 3-O-glucoside, peonidin 3-O-glucoside, and petunidin 3-O-glucoside were the pigments responsible for the purplish-red peel color. Transcriptomic analyses revealed that anthocyanin biosynthesis-related genes, from upstream (phenylalanine ammonia-lyase) to downstream (anthocyanidin 3-O-glucosyltransferase and anthocyanidin synthase), were upregulated with the accumulation of anthocyanins in the heterografted plants. At the same time, all these genes were also highly expressed and more anthocyanin was accumulated in self-grafted CS/CS samples compared with own-rooted graft-free CS samples, suggesting that self-grafting may also have promoted berry reddening in grapevine. Our results reveal global transcriptomic and metabolomic features in berry color regulation under different grafting conditions that may be useful for improving berry quality in viticulture.

## Introduction

Grafting has been practiced in horticultural plants for ~4000 years in China [1]; it establishes vascular continuity by joining the scion of one plant with the rootstock of another plant. The rootstock can benefit the scion plant by enhancing its resistance to biotic and abiotic stresses and improving desirable agronomic traits. Stem grafting in viticulture can be traced back to ~2500 years ago [2]. The practice of grafting in grapevine uses wild *Vitis* species as rootstocks, which provide advantages to the scion cultivar, including improvements in flowering time, berry quality, dwarfing, disease or pest resistance, and environmental adaptation [3]. Grafting connects two different genomes and introduces complex genomic regulation [3]. A subclade of *β*-1,4-glucanases contributed to grafting among a tomato scion, a *Nicotiana benthamiana* interscion, and an *Arabidopsis* rootstock by facilitating cell wall reconstruction [19]. In addition, heterografting a sweet orange scion to a *Poncirus trifoliata* rootstock was performed to investigate sRNA-mediated graft-transmissible epigenetic modifications in citrus [20]. Previous analysis showed that the rootstock can regulate the berry peel pigment of the scion cultivar [21], but the genetic basis and molecular mechanisms of the grafting effect on grape peel color are still unknown.

Grapevine coloring is a very important agronomic trait that is required for adaptation to the markets, including those for table and wine grapes. There are two types of grape berry color: peel color and flesh color. In general, most red grapes are pigmented in the peel, and the accumulation of anthocyanins in ripening grape berries occurs only in epidermal and subepidermal cells [4, 5]. Recent studies revealed that grape peel color was mainly determined by the composition and content of anthocyanins [6], and the relative proportion of anthocyanins in each grape variety is stable [7]. The anthocyanins in grapes include mainly delphinidin, petunidin, peonidin, and malvidin, which are composed of aminoglycosides or glycosides with acylation [16]. In grapevine, the contents of anthocyanins were lower in interspecific hybrids than in wild *Vitis* species and lower in table grapes than in wine grapes [17]. The biosynthesis of anthocyanins is also affected by light [8], temperature [9], moisture [10], mineral nutrients [11], cultivation measures [12, 13], growth regulators [14, 15], and other external factors.

Berry color is positively regulated by VvMYBA1, which binds to VvWDR1 and activates three promoters (*VvCHI3*, *VvOMT*, and *VvGST4*), whereas VvMYBC2-L1 negatively regulates this process by competing for the binding site with R2R3-MYB transcriptional activators or by repressing the expression levels of *VvOMT* and *VvGST4* [17]. Genomic structural variants showed that the QTL region (including *VvMYBA1*, *VvMYBA2*, *VvMYBA3*, and *VvMYBA4*) underlying berry color is hemizygous, and convergent evolution was associated with the origin of green coloration during grapevine domestication [18].

Crimson Seedless is an important grape cultivar with bright red berries and yellow flesh. This natural seedless late-ripening cultivar belongs to the European subspecies (*Vitis vinifera*), with thick fruit powder, hard, translucent flesh, and a high content of soluble solids. Understanding the effects of grafting on the anthocyanin synthesis pathway may be valuable for production of the Crimson Seedless cultivar and grapes in general.

In this study, we aimed at understanding metabolic differences and identifying significantly differentially expressed anthocyanin biosynthesis genes during berry development in heterografted(CS/101-14, CS/SO4, CS/110R), self-grafted (CS/CS), and graft-free (CS) plants. We studied the association of grafting, berry color, and metabolomic and transcriptomic profiles and revealed hub genes that play critical roles in anthocyanin biosynthesis under different grafting designs.

## Results

### Berry development and coloring

**Figure 1:**
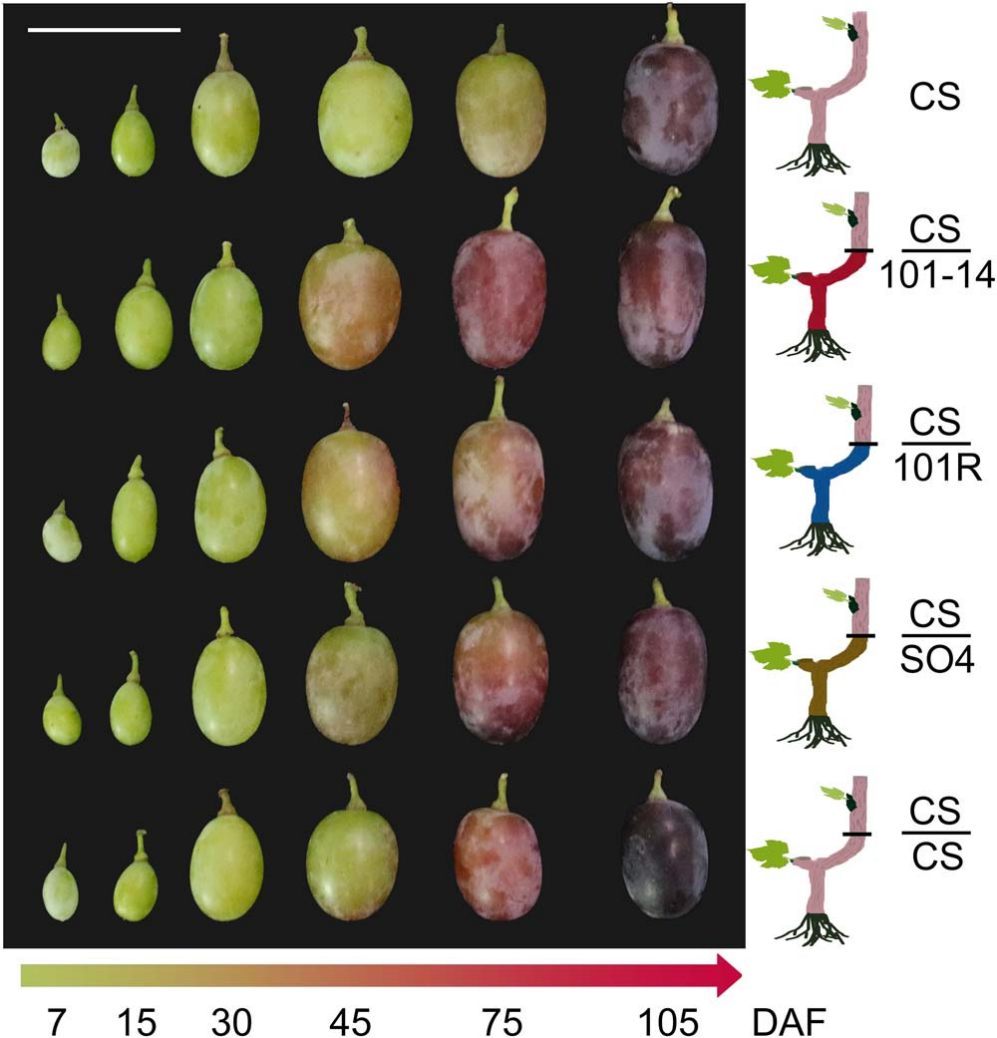
The grafting design and berry phenotypes of Crimson Seedless grapevine grafted onto different rootstocks. A schematic illustration of the grafting and phenotypes of grape berries at six developmental stages are shown.

We collected berry skin samples from Crimson Seedless graft-free, self-grafted, and heterografted plants. Berries of heterografted plants showed earlier initiation of berry coloring (38.5 days after flowering) and larger fruits than CS and CS/CS; the heterografted CS/101-14 plants showed the most obvious coloring (Figure 1). Three developmental periods were chosen based on phenotypic features and color changes. The first stage identified was 45 days after flowering (DAF), at which the skin of the three heterografted samples (CS/101-14, CS/SO4, and CS/110R) showed visible color but there were no differences in fruit size (Figure 1). At 75 DAF, all samples except for CS were undergoing veraison with berry skins turning red, and fruit size was larger in the heterografted samples (CS/101-14, CS/SO4, and CS/110R) than in self-grafted CS/CS and graft-free CS. At the final stage (105 DAF), all samples had finished veraison; CS/CS showed the darkest red color and the smallest fruit size, whereas the CS/101-14 plants had the largest fruit size. All three commercial rootstocks promoted fruit development, and the best rootstock appeared to be 101-14 (Figure 1).

**Figure 2:**
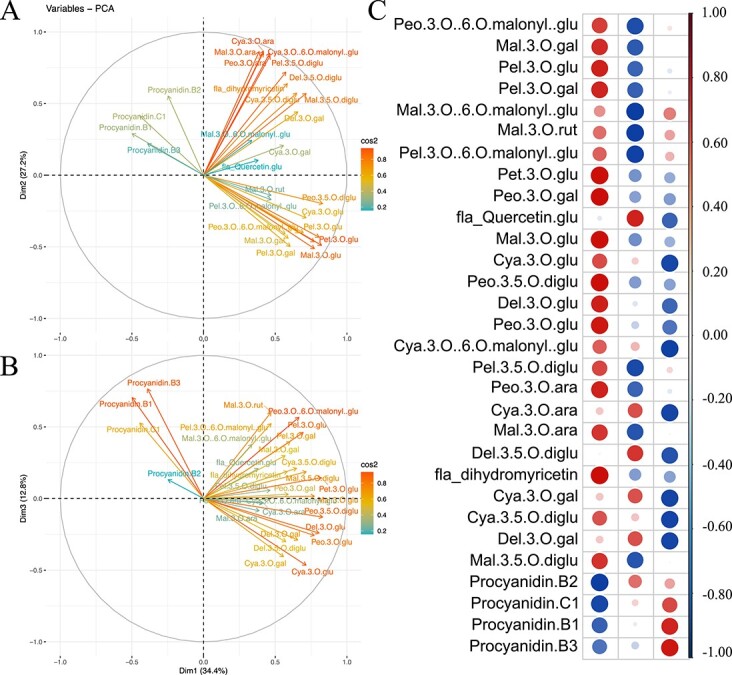
Unsupervised multivariate PCA analyses of metabolites and their association with berry color. (A and B) In the variable correlation plots of 30 metabolites, the distances between variables and the origin measure the quality of the variables on the factor map colored by cos2 value for PC1 & PC2 (A) and PC1 & PC3 (B). (C) A heatmap of cos2 values of variables on all dimensions.

### Metabolomic analyses detected metabolites related to anthocyanin synthesis

The metabolomes of 45 samples from five groups of grapevine plants (at three stages with three replicates each) were evaluated, and thirty kinds of metabolites related to anthocyanins were identified and classified into seven groups: cyanidin, procyanidin, peonidin, delphinidin, malvidin, pelargonidin, and petunidin (Figure 2). The content of 26 metabolites (86.7%) increased over the course of development and showed a significant correlation with the berry color phenotype (*P* < 0.05). The association of phenotype and metabolomics revealed a critical period associated with grafting (Figure 2). Unsupervised multivariate principal component analysis of the metabolites showed that the first three principal components explained 74.4% of the variance, and PC1 (34.4%) and PC2 (27.2%) described the compound distribution of the samples (Figure 2A and 2B). Compounds whose content increased were initially present at low levels, but their content gradually increased until it peaked at the third stage (105 DAF). Eighteen and five compounds, mostly colored anthocyanins, were explained better in PC1 and PC2, respectively (Figure 2A and 2B, Variable correlation >0.6). Three procyanidins and two cyanidins were separated by PC3 and decreased during development.

**Figure 3:**
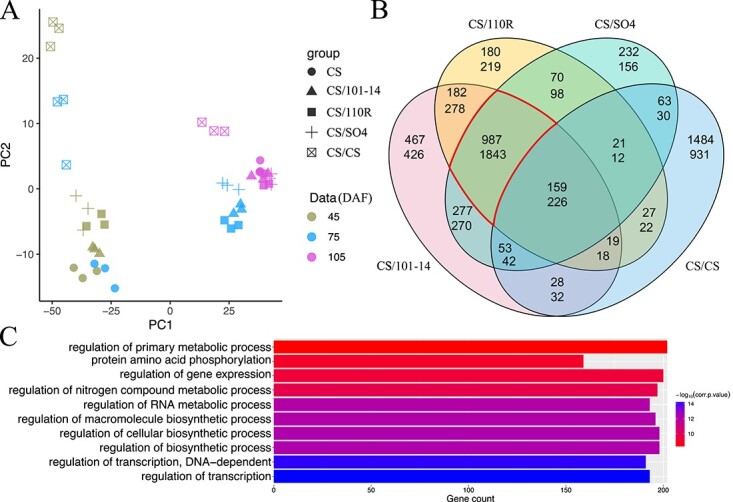
Variability of transcript levels among grapes grafted onto different rootstocks. (A) PCA results of the transcriptome data. (B) Overlap of DEGs in four grafted plant types compared with self-rooted plants; the upper and lower numbers indicate the numbers of up- and downregulated genes, respectively. (C) The first ten GO terms enriched in the common DEGs (highlighted in B) of the three rootstocks.

### An overview of the transcriptomic data

A total of 1.89 billion clean paired-end reads with a length of 150 bp were obtained from the RNA-seq dataset for 45 samples. All clean reads were mapped to the PN40024 reference genome (Ensembl; *V. vinifera* 12X). The uniquely mapped rate was >90% in all samples (Table S2). PCA was used to visualize and evaluate the overall differences in gene expression among different grafting combinations. The first two PCs explained 86.2% of the variation; the first PC (72.6%) separated all samples according to developmental stage, and the second PC (13.6%) separated the self-grafted CS/CS from the other four groups of samples (Figure 3A). According to the PCA, the distance between the three replicates of each sample type was close, suggesting that the data were highly consistent and of high quality. In addition, the five groups at 45 DAF showed a similar PC1 value from −25 to −50. At 75 DAF, the PC1 values of the three heterografted samples (CS/101-14, CS/SO4, and CS/110R) were around 25, whereas the PC1 values of self-grafted CS/CS and graft-free CS were less than −20. At 105 DAF, the three heterografted and graft-free samples gathered on the far right, whereas the self-grafted sample CS/CS had the most significant changes compared with 75 DAF. These results revealed that significant transcriptional differences between the three heterografted rootstock varieties and the two control samples occurred mainly at the second stage (75 DAF).

DEGs were identified based on |FPKM| > 1 and FDR ≤ 0.05. There were 11 972 DEGs in different stages compared with the self-rooted graft-free (CS) samples, representing 52.27% of the whole-genome transcripts. The number of DEGs in each group ranged from 550 to 4539. At 75 DAF, the DEG number was larger than at the other stages, consistent with the color phenotypes (Figure S1). The differences in gene expression observed in PCA were well supported by the DEG analyses (Figure 3A). According to the Venn diagram for 75 DAF, some differentially expressed genes were specific to CS/CS (1484 upregulated and 931 downregulated) or common to the three heterografted samples (987 upregulated and 1843 downregulated) (Figure 3B). We performed enrichment analysis to categorize the functions of the DEGs that overlapped in the three heterografted samples at 75 DAF. The results showed 136 and five enriched GO terms derived from the upregulated and downregulated DEGs, respectively. Sixty-six of the 136 upregulated terms belonged the BP category, in which “regulation of transcription”, “regulation of biosynthetic process”, “regulation of primary process”, “response to abiotic stimulus”, “response to carbohydrate stimulus”, and “response to mechanical stimulus” were the most enriched terms (Figure 3C, Table S3).

**Figure 4:**
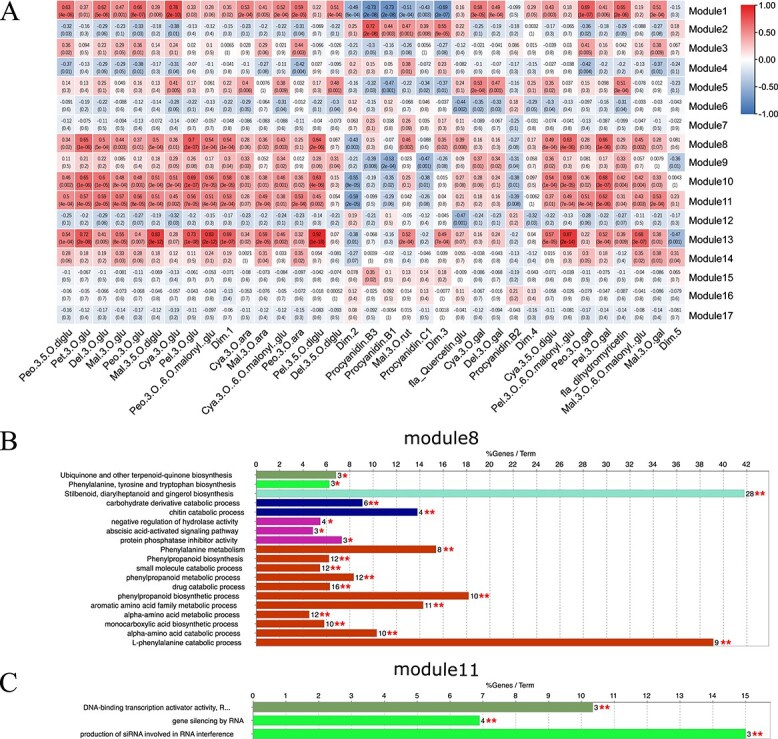
Module–anthocyanin association analysis. (A) Heatmap showing the correlation between modules and anthocyanins. Abbreviations and full names are given in Table S4. The GS-value between a given module and anthocyanins is indicated by the color of the cell and the text inside the cell (upper number is the value, and lower number is the P-value). Red and blue indicate positive and negative correlations, respectively. (B and C) GO-enrichment analysis of modules 8 (B) and 11 (C). ^*^P < 0.05, ^**^P < 0.01, and ^***^P < 0.001.

### Gene screening using WGCNA analysis

To classify co-expression modules and identify hub genes based on transcriptomic and metabolomic data, a weighted correlation network was constructed using 25 038 transcripts. A thresholding power of three was selected, which was the lowest power that properly fit the scale-free topological index, and 17 modules were revealed after the merged dynamic analysis (Figure S2A). The modules were sorted and numbered according to the gene number assigned to each module. Most of the genes (16 948) fell into the first module, modules 2–5 contained more than 500 genes, and the other 12 modules contained between 51 and 252 genes.

**Figure 5:**
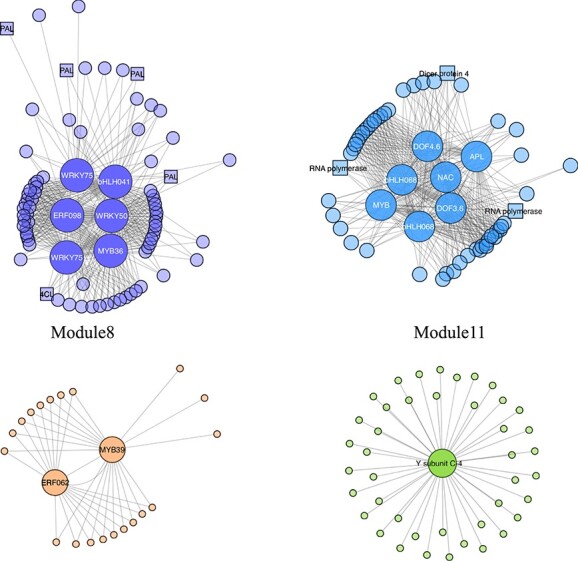
The correlation network in modules highly related to anthocyanin synthesis. The size of the node represents the number of connected genes. The transparency of the edges indicates the weight value between two genes.

The correlation coefficients between the modules and anthocyanin content varied widely, from −0.73 to 0.92. Four intriguing modules (modules 8, 10, 11, and 13) with GS-values greater than 0.5 in multiple compounds or PCs were screened, indicating that genes in these modules were significantly correlated with anthocyanin content. The biological functions of the intrinsic genes in the four modules were further analyzed (Figure 4A). First, because these four modules were in the same cluster, we analyzed the functions of all genes. The result revealed four terms related to chitin catabolic process; stilbenoid, diarylheptanoid and gingerol biosynthesis; abscisic acid binding; and phenylalanine ammonia-lyase activity (Figure S2B). Next, each module was checked, and the two modules we focused on were module 8 and module 11. Five KEGG terms and 14 GO terms were enriched in module 8, including five terms related to phenylalanine, such as KEGG:00940 (Phenylpropanoid biosynthesis) and GO:0009699 (Phenylpropanoid biosynthesis), showing that genes in this module participated in the synthesis and metabolism of compounds related to anthocyanins. In addition, KEGG:00945 (Stilbenoid, diarylheptanoid, and gingerol biosynthesis) and GO:0009738 (abscisic acid-activated signaling pathway) indicated that this module also plays other roles in berry development. Only 3 GO terms were enriched in module 11 (DNA-binding transcription activator activity, production of siRNA involved in RNA interference, and gene silencing by RNA), indicating that this module was mainly associated with siRNA activities. Connectivity, MM, and GS values of genes in each module were calculated and combined to identify the hub genes (Figure S3). Eighty-two, 22, 57, and 43 hub genes were identified in modules 8, 10, 11, and 13, respectively (Figure 5). Sixteen Hub-TF genes were detected and classified into eight TF families using PlantTFDB. Based on the Hub-TF genes and correlation network, we built and visualized a network highly related to anthocyanin synthesis (Figure 5).

### Expression patterns and validation of anthocyanin biosynthetic pathway genes

**Figure 6:**
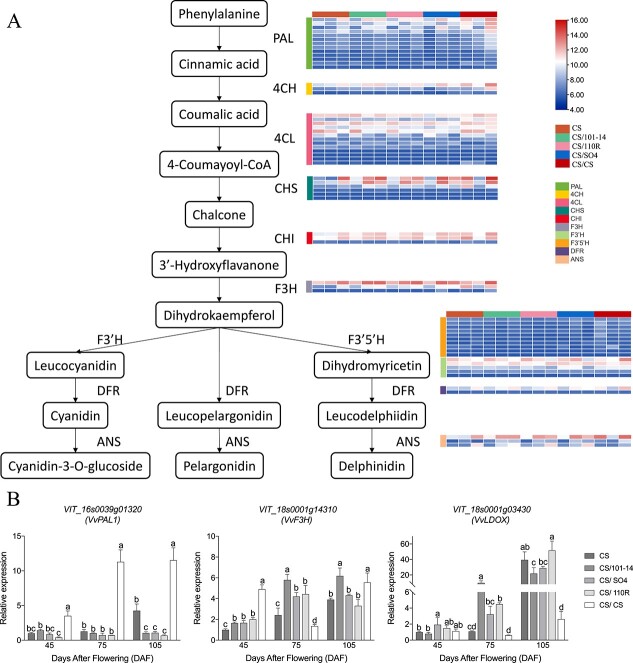
Transcript profiles (A) and RT-PCR data (B) for genes in the anthocyanin biosynthetic pathway. Grids with a color-scale from blue to white to red represent the gene expression of the DEGs from low to medium to high. PAL, phenylalanine ammonia-lyase; C4H, cinnamic acid 4-hydroxylase; 4CL, 4-coumarate CoA ligase; CHS, chalcone synthase; CHI, chalcone isomerase; F3H, flavanone 3-hydroxylase; F3′H, flavonoid 3′-hydroxylase; F3′5′H, flavonoid 3′,5′ hydroxylase; DFR, dihydroflavonol 4-reductase; ANS, anthocyanidin synthase.

We selected 19 genes in the anthocyanin biosynthetic pathway that belonged to 11 gene families. We found that all the genes were expressed differently in all samples (Figure 6, S4). During fruit coloring, most anthocyanin biosynthetic pathway genes were upregulated, with the highest expression levels at 105 DAF. However, each gene family had 2 (DFR) to 13 genes (PAL) with high expression levels. This indicated that although they may have similar functions, only a few genes were functional. Compared with self-grafted CS/CS and graft-free CS samples, genes in the heterografted group began to be upregulated later, especially *PAL*, *4CH*, *4CL*, *CHS*, *CHI*, and *F3H*, which participate in the synthesis of anthocyanin precursors in the anthocyanin pathway. A similar trend was observed in phenotypic processes and anthocyanin content, with higher gene expression in CS/CS at 105 DAF than in the other four groups (Figure 6, S4).

Finally, 19 DEGs, including four transcription factor genes, four genes of the phenylpropanoid biosynthetic pathway, and 12 genes of the flavonoid biosynthetic pathway, were selected to analyze their expression levels in all samples using RT-qPCR. The results demonstrated good consistency between RNA-seq data and RT-qPCR data, with a correlation coefficient of 0.9992 (Figure S5).

## Discussion

Grafting in grapevine production can improve the fitness and phenotypes of the scion, including berry quality, berry color, environmental adaptation, and disease resistance. According to our field observations, grafting of the CS scion onto the 101-14 rootstock had a positive effect on fruit coloring. However, the molecular mechanism underlying this observation was unknown. We combined berry color phenotypes with metabolomic and transcriptomic data at three stages of berry development of CS grafted onto four rootstocks, with own-rooted graft-free Crimson Seedless as the control. Heterografting upregulated the expression of genes involved in the anthocyanin biosynthesis pathway and promoted earlier reddening of berries in CS/101-14, CS/SO4, and CS/110R. TF factors are the hubs for regulation of early reddening. The self-grafted plants (CS/CS) also showed an earlier reddening phenotype, more anthocyanin content, and upregulation of genes in the anthocyanin synthesis pathway compared with graft-free plants (CS), suggesting that self-grafting alone may have positive effects on berry reddening in grapevine.

The pigments responsible for the purplish-red peel color of the Crimson Seedless cultivar included cyanidin 3-O-glucoside, delphinidin 3-O-glucoside, malvidin 3-O-glucoside, peonidin 3-O-glucoside, and petunidin 3-O-glucoside (Figure 2). In the samples of group CS/101-14, the content of anthocyanins significantly increased from 75 DAF, and at 105 DAF, the accumulation of anthocyanins was dramatically higher in all four grafted groups than in the CS group (Figure 1). These results showed that rootstock grafting can increase the anthocyanin content of grape berries and promote coloration and that grafting onto rootstock 101-14 can promote earlier anthocyanin accumulation in grape berries.

Previous studies have shown that the MYB-bHLH-WDR (MBW) regulatory complex coordinately activates multiple genes related to anthocyanins [39, 40]. In bright-colored fruits, genes encoding key enzymes downstream of the anthocyanin biosynthesis pathway, such as *DFR*, *ANS*, and *UFGT*, are often highly expressed [41]. MBW complexes consist of MYB TFs, basic helix–loop–helix (bHLH) TFs, and WD40 proteins, and they have been demonstrated to regulate the expression of anthocyanin-related genes [42]. In *Arabidopsis thaliana*, some MYB transcription factors such as TT2, MYB75, MYB113, and MYB114, some bHLH transcription factors such as TT8, GL3, and EGL3, and the WD40 repeat protein TTG1 can regulate the expression levels of several downstream genes, such as DFR, ANS, and UFGT, thereby affecting anthocyanin biosynthesis [42].

In this study, anthocyanin biosynthesis-related genes, from upstream (phenylalanine ammonia-lyase, cinnamic acid 4-hydroxylase, 4-coumarate CoA ligase, chalcone synthase, flavanone 3-hydroxylase, flavonoid 3′-hydroxylase, flavonoid 3′,5′-hydroxylase, flavonoid 3′-hydroxylase, flavonoid 3′,5′-hydroxylase, and dihydroflavonol 4-reductase) to downstream (anthocyanidin 3-O-glucosyltransferase and anthocyanidin synthase), were almost all upregulated with anthocyanin accumulation and berry reddening. However, all these genes were also highly expressed in CS/CS samples, and the results suggested that self-grafted plants may have an earlier response to fruit color-related metabolism. Differentially expressed MYB TFs, such as *MYB44* and *MYB4*, were hubs in the PPI network analysis. We predict that MYBs are the key regulators involved in anthocyanin pathways in the interactions between grapevine scions and rootstocks.

In apple, *CHS* is positively regulated by the expression of *MYB4* and *MYB5* [43]. *FcMYB1* in strawberry switches the accumulation of anthocyanins and flavonoids on and off [44]. However, the deletion of MYB *cis*-elements in the *CHS* promotor can produce white crabapple morphs [45]. In our PPI network analysis, *trans*-cinnamate 4-monooxygenase-like directly interacted with the MYB86 and MYB4 TFs; leucoanthocyanidin reductase 1 directly interacted with the MYBPA1 protein; flavonoid 3′,5′ hydroxylase, anthocyanidin 3-O-glucosyltransferase 2, and an MYC anthocyanin regulatory protein directly interacted with the MYB90 TF; and flavonoid 3′ hydroxylase directly interacted with MYB-related protein 308 and MYB-related protein 305.

The DELLA proteins positively regulate the biosynthesis of anthocyanins in *Arabidopsis*. They can directly interact with and sequester the AtMYBL2 and AtJAZ repressors, resulting in higher MBW complex activities [46]. A considerable number of anthocyanin repressors have been identified. In *Arabidopsis* seedlings, miR858 inhibits the expression of the anthocyanin repressor *AtMYBL2*, thereby positively regulating anthocyanin biosynthesis [47]. By contrast, miR858 inhibits the expression of *SlMYB7*-like in tomato to negatively regulate anthocyanin biosynthesis. Blocking miR858 function by ectopic expression of a small tandem target mimic of miR858 enhanced anthocyanin accumulation in tomato seedlings [48]. A high auxin concentration inhibits anthocyanin biosynthesis [49, 50]. A study of red-fleshed apple calli [51] demonstrated that Auxin Response Factor 13 (MdARF13) inhibited the biosynthesis of anthocyanin. This was achieved both by the direct binding of MdARF13 to the promoter of the ABP gene *MdDFR* to repress its expression and by the physical interaction of MdARF13 with the subgroup 6 R2R3-MYB activator MdMYB10 to destabilize the MBW complex. In the WGCNA network, the transcriptional repressor MYB36 TF was highly co-expressed with bHLH041 and WRKY75, regulating four PAL genes and one 4CL gene.

Dicer protein4 and two RNA polymerases were situated at the hub of the co-expression network and directly related to seven TFs, including two DOF TFs and two bHLH068 TFs. Dicer endonucleases participate in sRNA biogenesis and play an important role in plants; they can produce siRNAs that determine the specificity of the RNA-directed DNA methylation pathway. In soybean and peach, Dicer proteins were involved in the regulation of seed coat and fruit color through the production of siRNAs and significant increases in the transcription level of genes related to anthocyanin regulation, respectively [52, 53]. The importance of Dicer protein in anthocyanin metabolism was to be expected. Module 11 was significantly correlated with anthocyanin content and enriched in siRNA activities, and siRNAs play important roles in the regulatory networks between scions and rootstocks [3].

## Conclusions

In summary, comprehensive phenotypic, transcriptomic, and metabolomic analyses provided large-scale information on gene-metabolite regulatory networks related to anthocyanin synthesis. Our results reveal global transcriptional changes in grape peel color regulation under different grafting conditions and have implications for improving the production and breeding of grapevine.

## Materials and methods

### Plant materials and treatments

The grafting experiment was performed at the fruit quality control post base of the national grape industry technology system at the Anningqu comprehensive test field of the Xinjiang Academy of Agricultural Sciences, Xinjiang, China (87.28′ E, 45.56′ N). The site has a typical temperate continental climate with a frost-free period of 155–177 days; the first and the last frost occur from late September to mid-October and from the end of April to mid-May, respectively. The average minimum temperature in winter was −22.0°C, and the extreme minimum temperature was −40.0°C. The rootstock-scion combinations were planted on the same site in 2012 and began to bear fruit in 2014. The plant-row spacing was 1 × 3.5 m. The soil, fertilizer, and water management were normal, and the field management was consistent.

Scions were selected from thriving annual branches of Crimson Seedless self-rooted plants (CS). Three heterografted and one self-grafted combination were constructed: one grafted with 101-14 rootstock (CS/101-14), one with SO4 rootstock (CS/SO4), one with 110R rootstock (CS/110R), and one with Crimson Seedless rootstock (CS/CS) (Figure 1). Each grafting combination was performed with ten replicates. These three commercial rootstocks were selected from grape distant hybrids to improve disease and stress resistance and reduce the number of days until fruit ripening. A total of 50 berries were collected in randomized block designs for each grafting combination. The berry skins (10 g per sample) were extracted and sampled with three biological replicates at three stages: 45, 75, and 105 days after flowering (DAF) (Figure 1). The peels were carefully excised in the lab, then collected and frozen in liquid nitrogen. After being roughly ground, 45 samples were stored at −80°C for metabolome profiling, mRNA sequencing, and RT-qPCR validation.

### Metabolite identification and quantification

The anthocyanin profiles for each sample were obtained by the following three steps: grinding, extraction, and measurement. (i) A mixer mill (MM 400, Retsch) was used to crush the freeze-dried sample. (ii) Powder (50 mg) was placed in the extraction solution (methanol:water:hydrochloric acid, 799:200:1, v/v/v), vortexed and ultrasonicated for 10 min separately, and then centrifuged at 12,000 g and 4°C for 3 min; the supernatants were collected. The precipitate was processed again using the same method to fully extract the components. The supernatants were filtered (PTFE, 0.22 μm; Anpel) and combined for UPLC–MS/MS analysis. (iii) A UPLC (ExionLC AD) and tandem mass spectrometer (MS/MS) (QTRAP 6500+, N) were used to detect the anthocyanin contents. The integral peak area of all the detected samples was substituted into the linear equation of the standard curve for calculation. Finally, the absolute volume of the substance in the actual sample was calculated.

### RNA sequencing (RNA-seq) analyses and differentially expressed genes (DEGs)

Total RNA was isolated as follows: (i) Preheated fragmentation buffer and β-mercaptoethanol were added; (ii) chloroform/isoamyl alcohol (24/1) was added; (iii) the sample was shaken and centrifuged, and an equal volume of chloroform/isoamyl alcohol (24/1) was added to the supernatant, followed by centrifugation; (iv) step iii was repeated again, adding precipitant for precipitation and centrifugation and washing with ethanol to recover RNA. The resulting RNA was sent to Shanghai Personal Biotechnology Corp. Ltd for library preparation and RNA-seq.

High-quality clean reads were filtered from the raw reads using fastp [28] with default parameters. The clean reads were aligned to the *V. vinifera* reference genome (12X, http://plants.ensembl.org/Vitis_vinifera/Info/Index) using HISAT2 [29]. The mapped reads were assembled using StringTie [30] (http://ccb.jhu.edu/software/stringtie/), and the read count value of each mapped gene was calculated with HTSeq [31] as the original expression level of the gene. The count numbers were used as input data for variance stabilizing transformation (VST), a function integrated into DESeq [32], to produce transformed data on a log_2_ scale. Genes with |log_2_FoldChange| > 1 and P < 0.05 were considered to be differentially expressed.

Principal component analysis (PCA) was used to find associations in the metabolomic and transcriptomic data sets and to reveal specific metabolites and transcripts in categories [33–35]. The results were analyzed and visualized using R Studio software (https://www.rstudio.com/) with the two packages FactoMineR and factoextra.

### Enrichment analysis of gene function

We used ClueGO and CluePedia in Cytoscape [36] to classify genes functionally, and related terms with similar related genes were merged to reduce redundancy. The GO-term fusion function with default parameters was used to fuse similar items, and the threshold P < 0.05 with Benjamini and Hochberg’s FDR was used for hypergeometric testing. The Kappa scores were used to group terms using default parameters. Cytoscape and R were used to visualize the results.

### Identification of hub genes using WGCNA analyses

Weighted Gene Correlation Network Analysis (WGCNA) [37] was used to identify hub genes. First, cluster analysis was performed on the samples according to the expression levels of all genes. Then the TOMsimilarity module was used to calculate the co-expression similarity coefficient among genes. To reveal the functional connection of genes, the PickSoftThreshold function of the software package was used to select the parameters and perform the weighted calculation, which converted the expression similarity coefficient of the intermediate parameters into the connection between genes. The POWER value was selected when the correlation coefficient tended to be stable. According to the parameters selected above for network construction, a weighted co-expression network model was established to classify genes and divide thousands of genes into several modules. After a module was obtained, gene expression in the module was used to calculate the characteristic gene of the module, or the first main component of the module. The correlation between the characteristic gene of the module and the trait was calculated, including the correlation between the gene and the characteristic expression in the module (module membership, MM) and the correlation between each gene and the target trait (gene significance, GS).

Following a previous study [37], we used the passing threshold GS.abs >0.5 and GS.pvalue <0.001 to identify genes or modules that were significantly correlated with traits and the passing threshold GS.abs >0.5 and MM.abs >0.8 to identify the hub gene of each module. Transcription factor (TF) annotations were searched for the hub genes using PlantTFDB [38] (v5.0, http://planttfdb.gao-lab.org/). Cytoscape software was used to visualize the gene interaction network.

### RT-qPCR validation

The RNA used for RT-qPCR and RNA-Seq was extracted in the same batch. Primer3 (v4.0, https://bioinfo.ut.ee/primer3-0.4.0/) and NCBI Primer-BLAST (https://www.ncbi.nlm.nih.gov/tools/primer-blast/index.cgi) were used to design primers for RT-qPCR analyses (Table S1). The housekeeping gene *VvGADPH* was used to correct and compute the relative expression of candidate genes. The PCR assay was performed with the following conditions: 95°C for 2 min; 40 cycles at 95°C for 5 seconds, 60°C for 30 seconds, and 72°C for 10 seconds; and 72°C for 10 min.

## Acknowledgments

This research was financed by the National Natural Science Foundation of China (32160682, 31960575), the Young Doctor Fund of Gansu Province (2021QB-120), the Basic Scientific Research Funding Project of Tianshan Youth-Excellence Youth Project (No. 2020Q028), and the China Agriculture Research System of MOF and MARA. The central government guides local science and technology development special fund projects(2022) -Germplasm Innovation and Breeding Ability Improvement of Characteristic Fruit Trees.

## Author Contributions

Conceptualization: HZ, ZL, YZ, and FZ; Data curation: HZ, ZL, FZ, YP, XZ, XS, JW, WL, HX, and NW; Data analysis: HZ, ZL, WL, HX, and NW; Funding acquisition: HZ, FZ, YZ and MP; Methodology: HZ, ZL, and FZ; Project administration: HZ; Resources: FZ, ZL, XZ, and XW; Validation: HZ, ZL, and FZ; Writing, review, and editing: HZ, ZL, and YZ; Supervision: MP, XW, and YZ.

## Data availability

The RNA-Seq dataset from this study has been deposited at the NCBI under project number GSE184667.

## Competing interests

We declare that none of the authors have any competing interests.

## Supplementary data

Supplementary data is available at *Horticulture Research* online.

## Supplementary Material

Web_Material_uhac103
