# Supplementary material for: Metabolomic and transcriptomic analyses reveal the effects of self- and hetero-grafting on anthocyanin biosynthesis in grapevine
Source: Hortic Res. 2022 May 17;9:uhac103. doi: 10.1093/hr/uhac103 (PMC9251602; doi:10.1093/hr/uhac103)
Supplement: Web_Material_uhac103 [file web_material_uhac103.zip › FigS.docx]

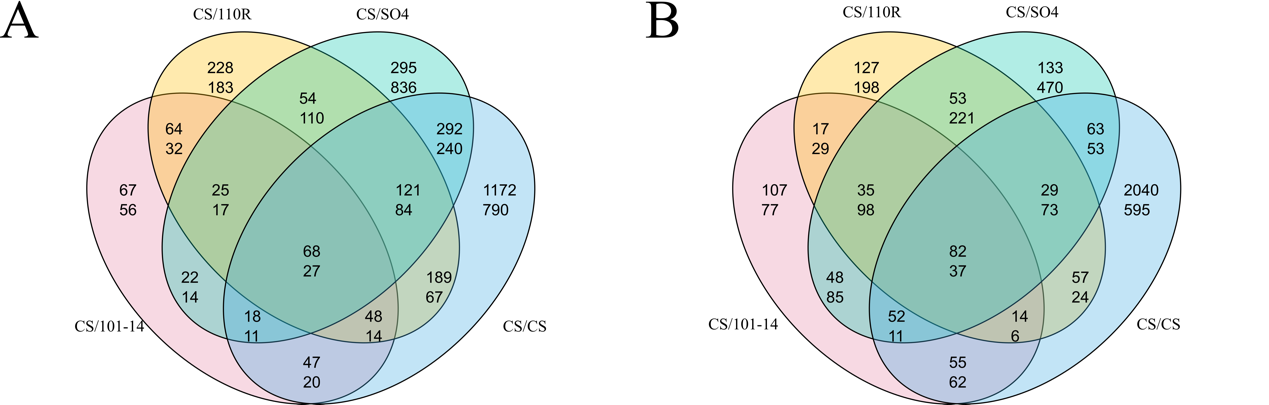


FigureS1 Veen plots showing the overlap of DEGs in 2 stages: 45 DAF(A) and 105DAF (B).
Overlap of the DEGs in 4 rootstocks compared with self-root. Upper number is the gene number of up- regulated and the lower number means down-regulated.


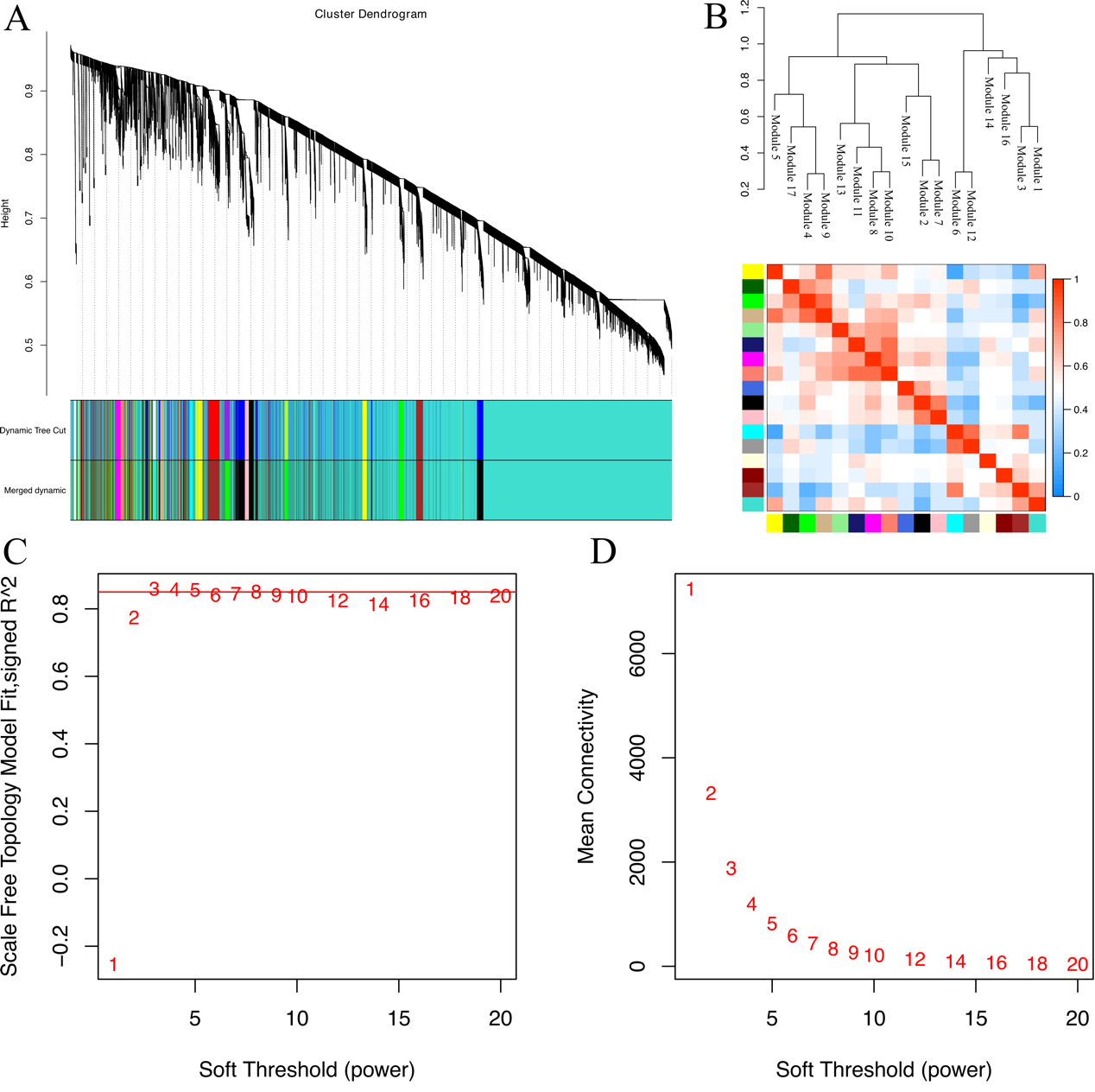


FigureS2 Weighted-gene co-expression network
A Hierarchical cluster dendrogram constructed by WGCNA, on which each leaf represents a gene. 17 merged modules (based on a threshold of 0.20) identified by weighted-gene co-expression network.
B. Module cluster dendrogram and module adjacency heatmap. Cluster dendrogram of module eigengenes. Branches of the dendrogram group together eigengenes that are positively correlated.


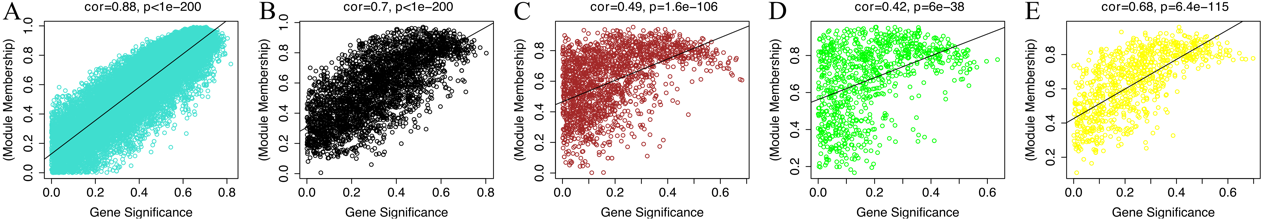


FigureS3 Relationship between module membership (MM) and gene significance (GS) Scatterplots show the relationship between GS and MM in first 5 modules(A-H: Module 1-5). Illustrating that gene highly significantly associated with a trait are often also the most important (central) elements of modules associated with the trait.


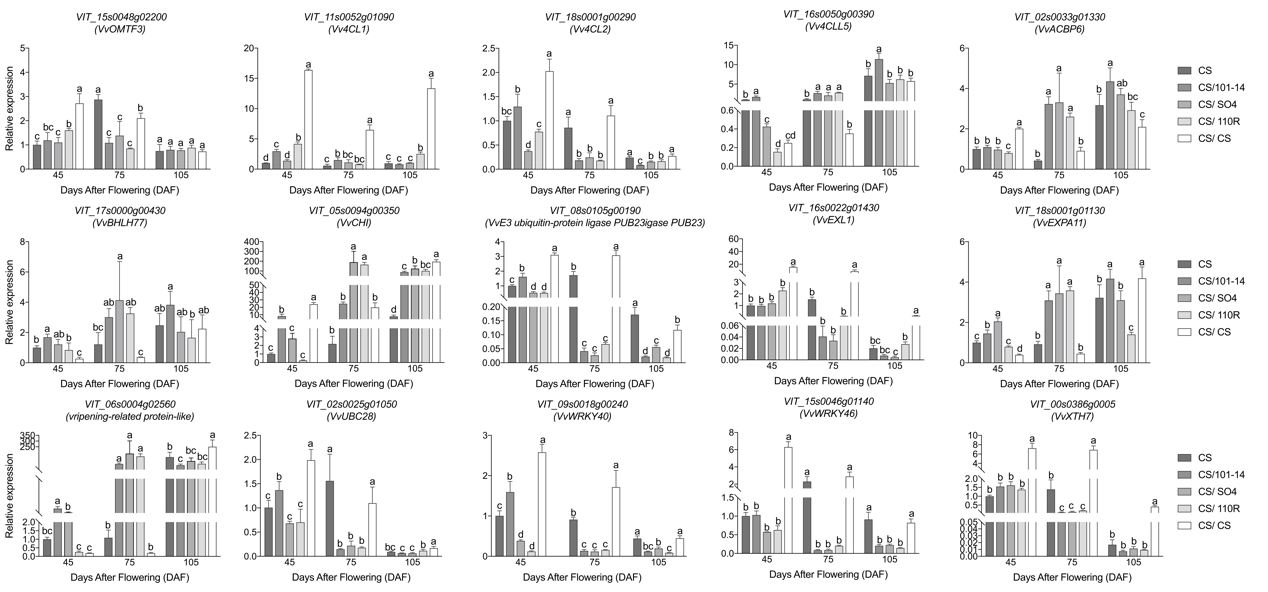


FigureS4 Expression patterns of 15 DEGs in three stages of grape with self-root and grafted on 4 rootstocks. Lowercase letters indicate significance at the 0.01 levels.


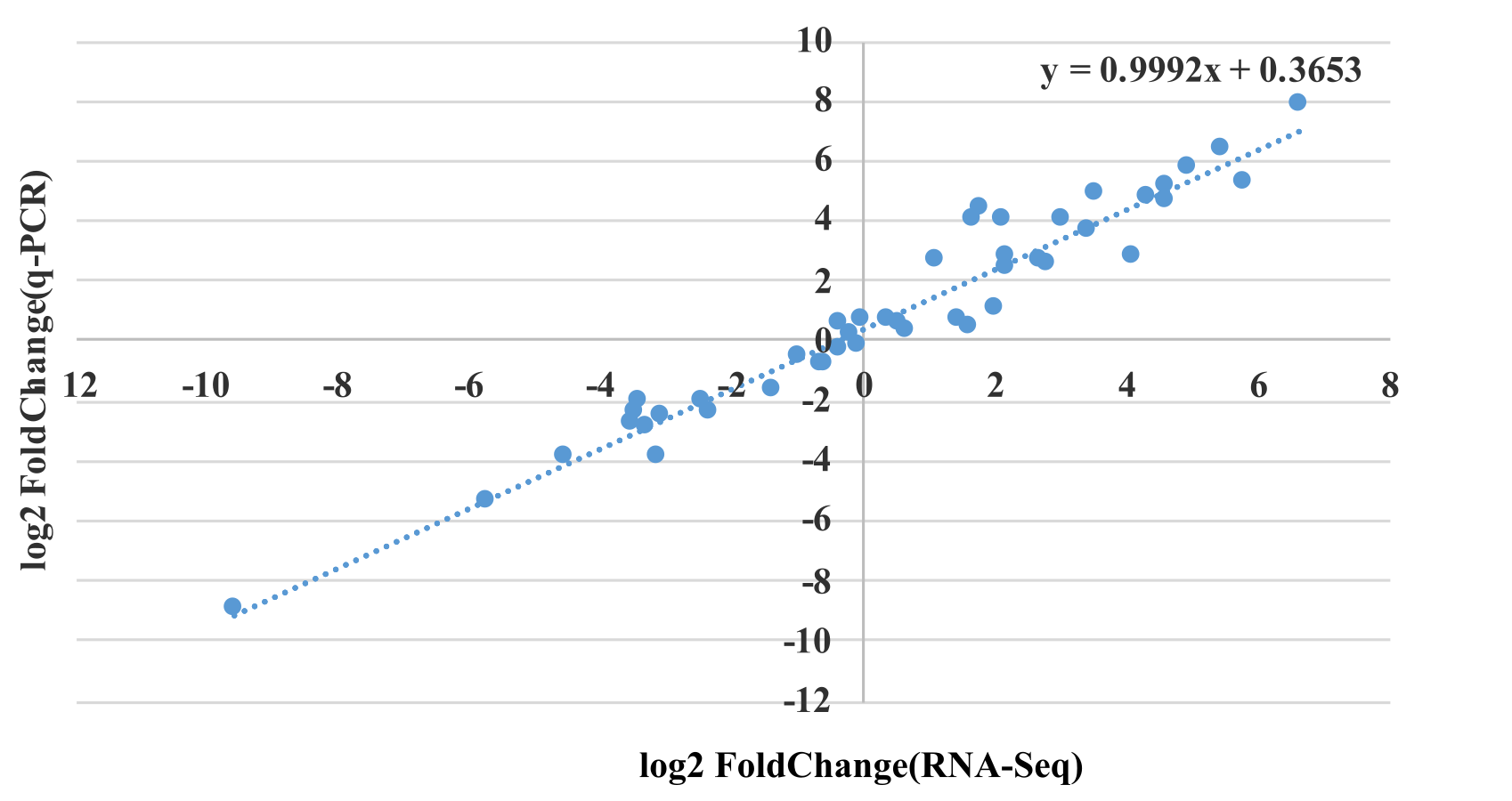


FigureS5 The correlation coefficient diagram of RNA-Seq data and RT-qPCR.
